# Visualizing uncertainty in habitat suitability models with the hyper‐envelope modeling interface, version 2

**DOI:** 10.1002/ece3.4720

**Published:** 2018-12-18

**Authors:** James Graham, Melissa Kimble

**Affiliations:** ^1^ Humboldt State University Arcata California

**Keywords:** Bezier curves, habitat suitability modeling, hyper‐envelope modeling interface, species distribution modeling, species niche, uncertainty

## Abstract

Habitat suitability models (HSMs) are popular and used for a wide variety of applications but most do not include analysis of the uncertainty of the model outputs. Additionally, some overfit the data and few allow the ability to fill data gaps with expert opinion. HEMI 1 addressed issues with overfitting data and allowed models to incorporate both occurrence data and expert opinion. HEMI 2 improves on HEMI 1 with a simplified interface and the ability to inject random noise into occurrence locations and environmental variable values to generate uncertainty maps. HEMI 2 uses Monte Carlo methods to perform uncertainty, validation, and sensitivity testing and generates mean and standard deviation habitat suitability maps.

## 1 INTRODUCTION

Ecological niche models, also known as habitat suitability models (HSMs), or species distribution models (SDM), have been used to generate maps of potential species habitat (Elith et al., 2011; Franklin, 2010). These models have also been used for conservation and restoration ecology (Elith & Leathwick, 2009), identifying potential hot spots for invasive species (Evangelista et al., 2008; Jarnevich & Reynolds, 2011), and locating rare and endangered species (Guisan et al., 2006).

HSMs attempt to represent the correlation between environmental variables and existing species’ locations (occurrence points). The environmental variables, also known as covariates, can be represented by a wide variety of parameters including temperature, precipitation, elevation, and slope. HSMs typically generate a georeferenced image, referred to as a raster where each pixel within the raster contains a value from 0 (unsuitable habitat) to 1 (suitable habitat) (Franklin, 2010). The occurrences and environmental variables utilized can contribute uncertainty to HSMs (Barry & Elith, 2006).

### 1.1 Occurrence uncertainty

All occurrence data contain uncertainty from a variety of sources of error and inherent variability in the modeling process. Because occurrences are observations of a species’ presence at a specific point in time, the uncertainty can include the following: species identification, coordinate uncertainty, and temporal uncertainty (Barrows, Preston, Rotenberry, & Allen, 2008; Elith, Burgman, & Regan, 2002; Peterson et al., 2011). Coordinates collected with a geographic positioning system (GPS) handheld will have an error between 3 meters and 10 s of meters (USDOD, 2008; Wing, Eklund, & Kellogg, 2005) which will have little impact on most HSMs. However, other coordinates, such as those computed from natural history collections, may be off by 10 s of kilometers (Wieczorek, Guo, & Hijmans, 2004) which can increase the uncertainty of model outputs (Moudrý & Šímová, 2012).

HSMs can underrepresent the potential distribution of a species if the occurrence data that is used to create the model do not represent the full environmental range of a species (Thuiller, Brotons, Araújo, & Lavorel, 2004). This can be caused by a lack of observations of a species’ presence, or to barriers, such as a mountain range, that have prevented the species from dispersing to otherwise suitable habitat (Pearson, 2010). Sampling bias occurs when some areas are sampled more than others (Anderson & Gonzalez, 2011). Additionally, occurrence data may be biased by effects such as sampling more frequently near roads, which can bias the estimated niche (Anderson & Gonzalez, 2011; Kadmon, Farber, & Danin, 2004; Phillips et al., 2009).

### 1.2 Environmental variable uncertainty

Environmental variables come from a wide range of sources and contain varying levels of uncertainty. Environmental variables used in HSMs are typically represented by grids of pixels or raster data that contain measurements of that environmental variable. These variables can include, but are not limited to the following: climate (temperature, precipitation, humidity), topography (elevation, slope, aspect, roughness), and proximity (distance to streams, distance to roads; Franklin, 2010).

Each pixel of an environmental variable contains a single value representing a complex spatial area on the ground (Pixel Mixing; Cracknell, 1998). Further, one pixel cannot represent microclimate temporal dynamics (Kearney et al., 2014). Small habitats, such as refuges for species, may therefore be underrepresented, or not represented at all (Gottschalk, Aue, Hotes, & Ekschmitt, 2011). Environmental variables such as temperature and precipitation can also represent periods of time that may not correlate well with species establishment (Roubicek et al., 2010). These sources of error can contribute to a misleading sense of confidence in model outputs when model uncertainty is not presented (Gould et al., 2014).

Information on uncertainty in environmental variables may be available only as summary information across the entire environmental variable, or not at all. This error may be available as a range (Hijmans, Cameron, Parra, Jones, & Jarvis, 2005), squared‐error terms (Hutchinson et al., 2009), or more complex distributions (Kimble, 2016).

### 1.3 Visualizing uncertainty with Monte Carlo methods and random noise injection

Monte Carlo (MC) methods are commonly used when evaluating HSM uncertainty (Elith et al., 2002). Cross‐validation, or repeatedly subsampling occurrence data into test and training data sets, is a common method to evaluate a model's robustness against its occurrence data. Recently, Gould et al. (2014) created visualizations of the impacts of spatial uncertainty by injecting random noise into the occurrences of *Anthochaera paradoxa* (yellow wattlebird). Maps were created by finding the proportion of pixels in the output that predicted potential species habitat in 100 models. A similar process was used to create visualizations of the impact of uncertainty in climatic environmental variables on predicted species distributions (Gould et al., 2014).

### 1.4 HEMI 1

The first version of the Hyper‐Envelope Modeling Interface (HEMI) created HSMs using three‐dimensional response surfaces for each pair‐wise combination of environmental variables (Graham et al., 2013). However, these surfaces were difficult for users to interpret and performance issues limited the number of environmental variables HEMI 1 could support.

## 2 HEMI 2

The following were the goals for HEMI 2:

1. Represent the fundamental response of a species to each individual environmental variable by fitting the model to occurrence data and allowing the user to modify the response curves to mitigate for gaps in the occurrence data.
2. Resolve the performance issues from the first version of HEMI and thus allow a larger number of environmental variables to be used.
3. Expand the options to include uncertainty information based on error in the occurrence data and environmental variables.
4. Provide sensitivity testing for model parameters and validation testing.

The ability of HEMI 2 to model four tree species in North America and address known gaps in occurrence data were shown by Kimble, 2016. Kimble also evaluated HEMI 2’s ability to inject random noise into occurrence data and environmental variables and produced uncertainty maps for these tree species. This study covers the remaining two HEMI 2 goals: to evaluate HEMI 2’s operation with synthetic data sets with known sources of uncertainty and to evaluate model robustness with validation and sensitivity testing for model parameters.

### 2.1 HEMI 2’s modeling approach

**Figure 1:**
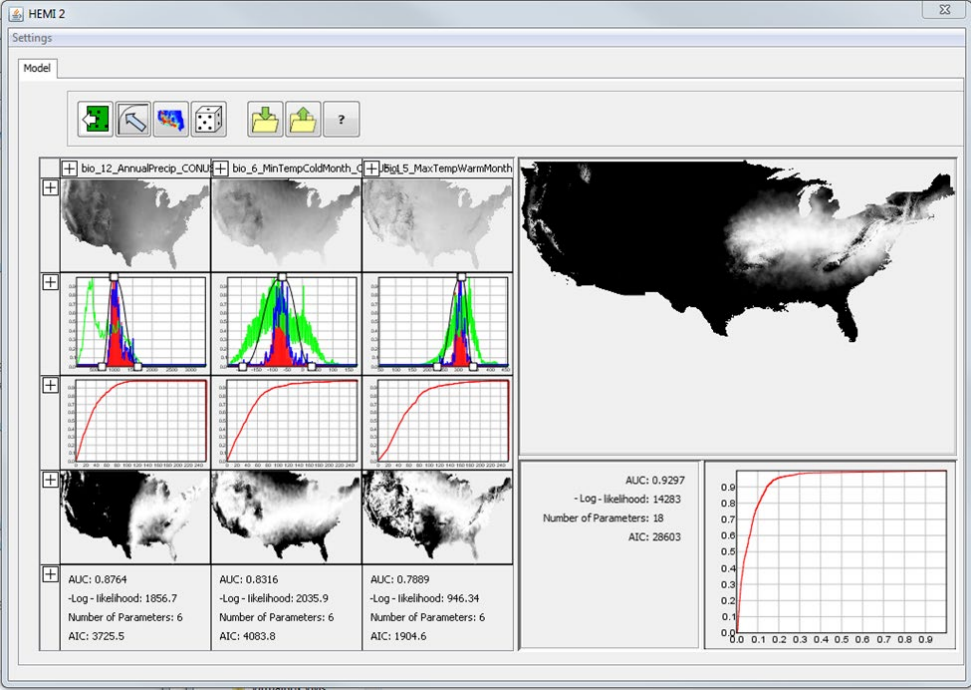
A screen capture of the main HEMI 2 window with three environmental variables using occurrences for Ohio Buckeye in the continental United States. Data for each environmental variable appear in the columns on the left while the final model and its associated receiver operator characteristic curve (ROC curve) are on the right. For each environmental variable, each column contains, from top to bottom: An image of the variable, a model graph, a ROC curve, a map of habitat suitability based on that variable, and model statistics. The model graph contains a green histogram for all the environmental variable values, a red histogram for the environmental variable values at the occurrences, and a blue histogram for the red histogram divided by the green histogram. The black line represents the model fitted to the specific environmental variable

In the main HEMI 2 window, each environmental variable is represented by a model graph, which is the software's representation of the species’ response to that environmental variable. The graphs cover the range of each environmental variable on the horizontal axes. If we define *z* to be one of the environmental varaibles, then *f*(*z*) represents the frequency of all the environmental variable values (Figure 1, green line), *f*_1_(*z*) represents the frequency of environmental values at just the occurrence locations (Figure 1, red line), and *f*_1_(*z*)/*f*(*z*) represents the division the two (Figure 1, blue line) (Elith et al., 2011). This division seeks to remove the bias from under‐ or overrepresented environmental values (Peterson et al., 2011). Frequencies are represented by histograms with 256 bins and are rescaled such that their maximum value on the *y*‐axis is 1 to aid in visualizing the histograms together.

**Figure 2:**
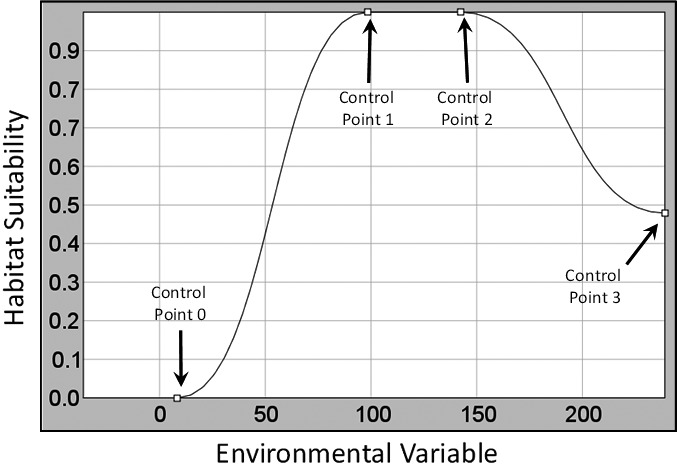
Each model is defined by four control points

While HEMI 2 can generate models using continuous or categorical variables, this study focused on continuous variables. An online tutorial is available that includes the use of categorical variables in HEMI 2 (http://gsp.humboldt.edu/HEMI2). Continuous models fitted to each environmental variable are represented by three Bezier curves (Figure 2). The control points can be manually adjusted by the user or automatically fit by HEMI 2. The first control point (Control Point 0) is restricted to the left and bottom sides of the graph. If this control point is on the left side of the graph, then our minimal environmental condition for this species was not within the sampled area. In other words, the environmental niche of the species may have been truncated. If this same control point is on the bottom side, then all environmental variable values to the left of this point were not considered suitable habitat for the species. The same restriction applies to the last control point (Control Point 3), but to the bottom and right sides, respectively. The remaining control points (1 and 2) can move anywhere within the graph as long as Control Point 0 is to the left of Control Point 1, Control Point 1 is to the left of Control Point 2, and Control Point 2 is to the left of Control Point 3.

### 2.2 Fitting the model

Models are fit automatically by placing a grid of possible positions for the control points over the graph and then trying all possible combinations of positions for the control points on the grid, and then selecting the positions that provide the maximum likelihood. The grid is created with 10 rows by 10 columns over the entire range of the environmental variable (*x*‐axis) and from 0 to 1 for the habitat suitability (*y*‐axis). The process is then repeated recursively with grids around the selected position. The number of grids and their width can be set by the user.

The models are fit to *f*_1_(*z*)/*f*(*z*) by default as this compensates for small numbers of occurrences because of environmental variable ranges that are underrepresented. Similarly, fitting models to fit to *f*_1_(*z*)/*f*(*z*) will keep environmental variable ranges that are overrepresented from dominating a model when they only include only a small number of occurrences as. If desired, the models can also be fit directly to *f*_1_(*z*).

Maximum likelihood can be computed by multiplying together the probability of each occurrence, given the model, together as in:

$$L\left(\Theta |x\right)=\prod_{i=0}^{n}p\left(x_{i}|\Theta \right)$$

Where L is the likelihood of the model Θ given a dataset *x*,* p* is the probability of a specific occurrence given the model Θ, and *n* is the number of occurrences. By taking the natural log of the equation above, we obtain the following:

$$\text{ln}\left(L\left(\Theta |x\right)\right)=\sum_{i}^{n}\ln\left(p\left(x_{i}|\Theta \right)\right)$$

This results in an increasing number of calculations and can be time‐consuming for large datasets. We can take advantage of the histograms in HEMI 2 to reduce the number of calculations with:

$$\ln\left(L\left(\Theta |x\right)\right)=\sum_{j}^{m}\ln\left(p\left(h_{j}|\Theta \right)\right)C_{j}$$

where *m* is the number of bins in the histogram, *h_j_* is the environmental variable value that corresponds to bin *j*, and *C_j_* is the number of occurrences in bin *j*. *p*(*h_j_*|Θ) is computed by selecting the appropriate model value for the bin *h_j_* and converting this value to a probability by dividing it by the area under the model. The negative of the result can then be used directly to compute the likelihood portion of Akaike information criterion (AIC, Akaike 1974 ).

For continuous data, each model has four control points with the first and last having one degree of freedom and the middle two having 2 degrees of freedom. This gives six estimated parameters for each model. Since all of the models have the same number of estimated parameters, or control points, the model with the maximum likelihood will also have the minimum AIC.

$$\text{AIC}=2k-\ln\left(L\right)$$

Using histograms greatly improves the speed of finding the most parsimonious model fit and makes the time to fit the model the same regardless of the number of occurrences provided. The trade‐off is a small amount of difference between the histogram‐computed AIC values and the traditionally computed AIC values from direct environmental variable values. This difference is introduced when environmental variables are quantized into histogram bins. However, since HEMI 2 uses 256 bins, the difference is less than half of a percentage point.

HEMI 2 also computes receiver operator curves (ROC) and area under the curve (AUC) (Fielding & Bell, 1997) performance metrics. Likelihood (effectively AIC) was preferred for fitting the model because it selects more parsimonious models (Burnham & Anderson, 2002).

### 2.3 Monte Carlo features

Random noise injection, sensitivity testing, and cross‐validation testing were implemented in a single Monte Carlo feature. Random noise can be injected into occurrence data and/or the environmental variables, and the models can be fitted repeatedly to characterize the impact of noise on the models and resulting habitat maps. Cross‐validation and sensitivity testing can also be run independently or with noise injection. This allows the flexibility to evaluate each of the Monte Carlo features independently or to examine the combined effects of multiple areas of uncertainty. Outputs of Monte Carlo runs include frequency histograms for area under the curve (AUC) and AIC values, jackknife results which show the AIC and AUC values for each combination of environmental variables, and results for each iteration of the model.

**Figure 3:**
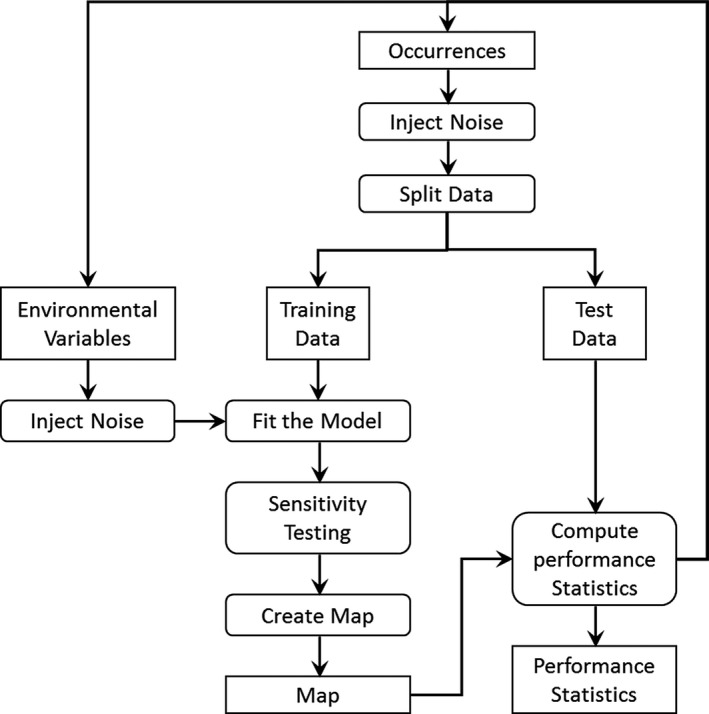
Diagram of HEMI 2’s Monte Carlo feature. The process begins at the top with injecting noise into the occurrences and environmental data. The occurrences are then split into test and training data sets with the training data used to fit the model against the environmental variables. Sensitivity testing injects noise into the model before the final habitat map is created. Then, the test data are used to compute performance statistics which are saved for each run. The process repeats a specified number of iterations

When the locations of occurrences are uncertain, random values are drawn from a noise distribution specified by the user and then added to each occurrence coordinate. For environmental variables, values are drawn from a specified distribution and then added to the pixels within the environmental variable raster. For regions where the uncertainty may change spatially, such as a desert region with plateaus and canyons, the parameters for the random distribution can be specified with rasters. After noise injection, the occurrences are divided into test and training data, the model is fit, sensitivity analysis is performed by injecting noise into the estimated parameters for the model (i.e., the control point positions), and then, performance statistics are computed based on the test dataset (Figure 3). The user may select any combination of steps and set the number of iterations for HEMI 2 to execute. When all iterations have been completed, summary performance statistics are made available on a web page with maps representing the minimum, maximum, and mean habitat suitability maps. A map of the standard deviation for each pixel of the habitat maps is also provided to evaluate areas where uncertainty is higher than others. HEMI 2 also executes a jackknife operation to provide performance metrics for each combination of environmental variables. Charts are also included in the output showing the response curves with minimum, maximum, and mean values and a 95% confidence interval. Histograms of the log‐likelihood, AIC, and AUC values are provided with charts of the cumulative means and standard deviations for AIC and AUC to help determine the number of runs that are required for the model to reach a stable state.

## 3 CREATING HABITAT SUITABILITY MAPS FROM SYNTHETIC DATA

The first step in our analysis was to show that HEMI 2 could produce an accurate habitat suitability map from synthetically created occurrence data and environmental variables. Using synthetic data within a habitat model has the advantage of allowing the modeler to compare the model's performance with an expected result (McCune, 2006).

**Figure 4:**
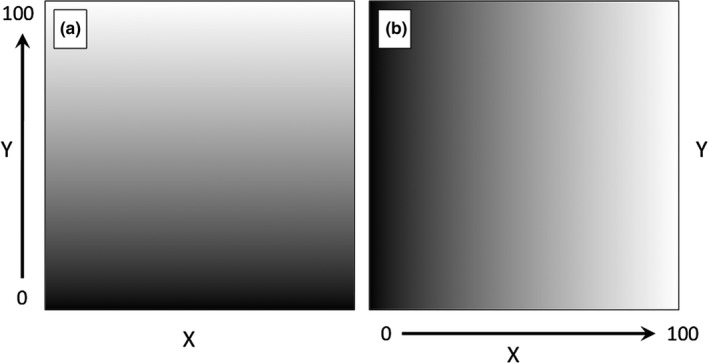
Synthetic environmental variables representing a range of a fictitious environmental variable with a range in the study area from 0 to 100. Variables are named “BottomToTop” (a) and “LeftToRight” (b) based on the direction of change for the variable

To begin, two synthetic environmental variables were created with a uniform range to simplify interpretation of model results. Each variable represented a measured environmental value that ranged uniformly from 0 to 100; where one changed in the Y direction (Figure 4a) and the other changed in the X direction (Figure 4b).

**Figure 5:**
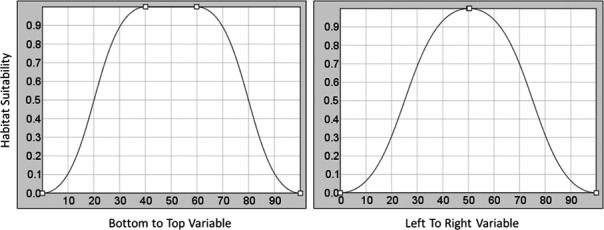
The synthetic response curves for the “BottomToTop” variable and the “LeftToRight” response variable with the “best” habitat for the species being at approximately 50 with poor habitat at 0 and 100

We adjusted response curves to represent the response of the hypothetical species to each synthetic environmental variable, effectively describing the environmental range of the species. The response curve for the “BottomToTop” variable was chosen to be relatively wide while the response curve for the “LeftToRight” variable was made to be relatively narrow (Figure 5).

**Figure 6:**
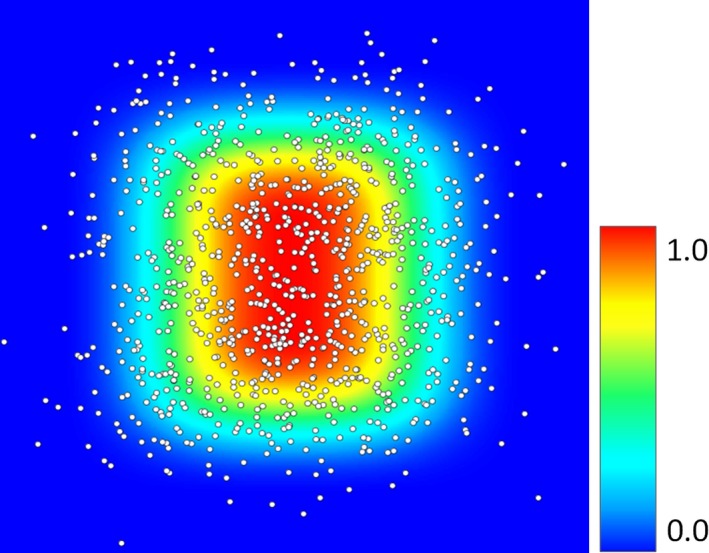
A simulated habitat map with occurrences added based on the probability of survival from the underlying pixel for the habitat map

A synthetic habitat map was then produced using the value of each corresponding pixel from the environmental values to obtain a habitat suitability value from each response curve and then multiplying the values together. This map represents the environmental range of the species. Species suitability to the environment was then simulated by uniformly distributing random occurrences across the synthetic habitat. Occurrences were removed if their corresponding value for habitat suitability was below a random value generated from a uniform distribution from 0 to 1.0 (Figure 6).

**Figure 7:**
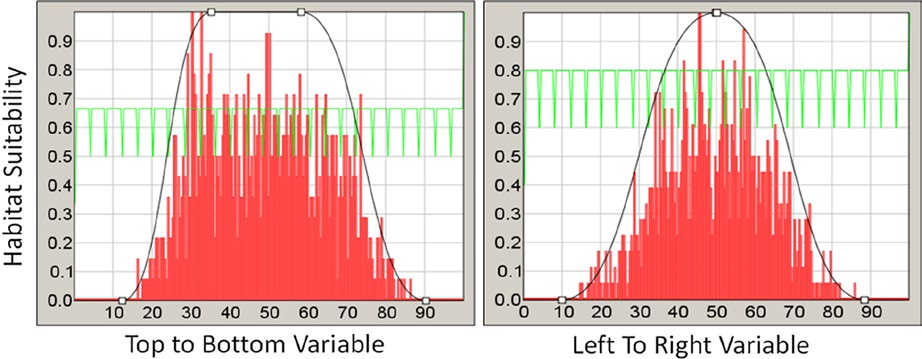
Modeled response curves. The green histogram graph is a histogram of environmental variable the full area while the red is a histogram of the environmental variables at the occurrences. Note that the curve on the left is skewed to the left because the random selection of occurrences based on the habitat model happened to be skewed to the left

**Figure 8:**
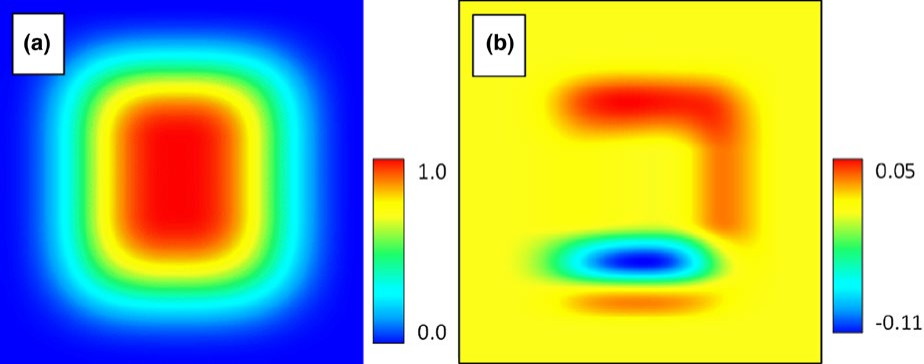
Habitat map generated by HEMI 2 from the modeled response curves (a) and the result of subtracting the original habitat map from the predicted map produced by HEMI 2 (b)

We then ran HEMI 2 against these filtered occurrences and the original synthetic environmental variables to produce habitat suitability models for each environmental variable (Figure 7). These models were used to produce a predicted habitat suitability map based on the occurrences (Figure 8a). Subtracting the HSM developed from the response curves from the original habitat map resulted in a maximum difference of 11%. The skewing of the response curve for the BottomToTopResponse curves shown visible as the difference of 11% toward the bottom of the image (Figure 8b).

## 4 Modeling the uncertainty of occurrence data

**Figure 9:**
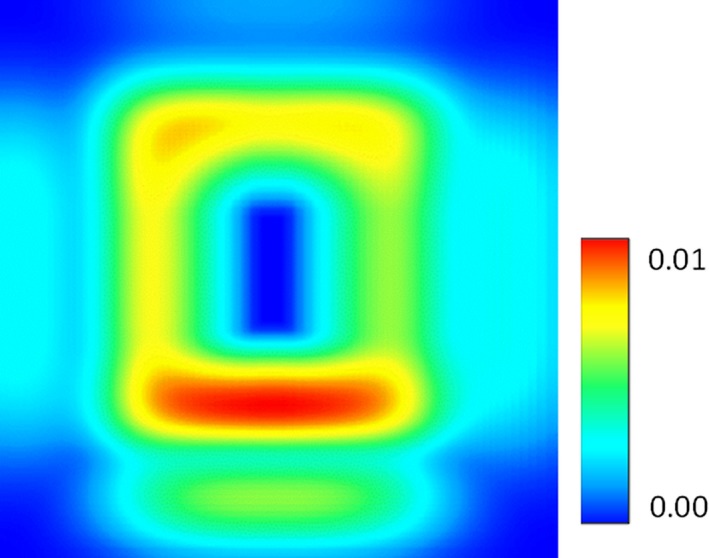
Uncertainty map of the standard deviation of 100 model runs with noise injected into the occurrence coordinates

**Figure 10:**
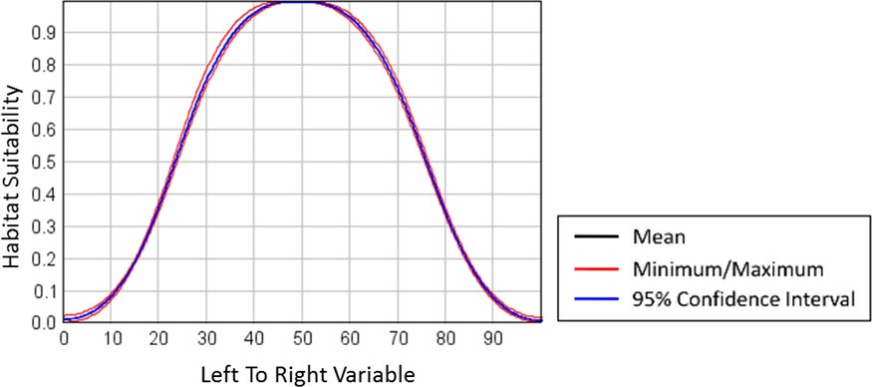
Response curve for the LeftToRight environmental variable showing the mean, minimum, and maximum curves for all model runs. The 96% confidence interval has little difference from the mean and is covering it in this graph

Random noise was injected into the synthetically created occurrence locations by randomly altering the original occurrence locations by a standard deviation of 1% (10 units). We assumed that the study area is a small region that is 1 km in each direction and contains a microhabitat similar to the one modeled in the previous section. In this case, a standard deviation of 1% would be equivalent to 10 m, which is typical for a handheld GPS (USDOD, 2008; Wing et al., 2005). The model was run 100 times and resulted in a mean AIC of 26,423 with a standard deviation of 14. The mean AUC value was 0.78 with a standard deviation of less than 0.001. HEMI 2 also produces maps of the standard deviation (Figure 9) and response curves showing the mean, minimum, maximum, and confidence intervals (Figure 10). The maps can be used to evaluate the spatial distribution of high or low confidence within our models, while the response curves characterize the variance within our models for each environmental variable.

**Figure 11:**
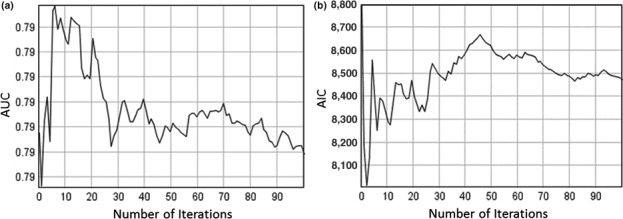
Graphs showing that the running AUC means (a) and the running AIC means (b) (vertical axis) have effectively stabilized after 100 model runs (horizontal axis)

HEMI 2 produced histograms of the AIC and AUC values and graphs of the cumulative mean value for AIC and AUC (Figure 11) for all model runs. The histograms should approach a normal distribution when sufficient runs have been completed and the cumulative AIC and AUC curves should show that these values are stabilizing over time. The histograms and performance metrics for each run are available on the HEMI 2 Web site (http://gsp.humboldt.edu/HEMI2 ).

## 5 MODELING UNIFORM UNCERTAINTY IN ENVIRONMENTAL VARIABLES

**Figure 12:**
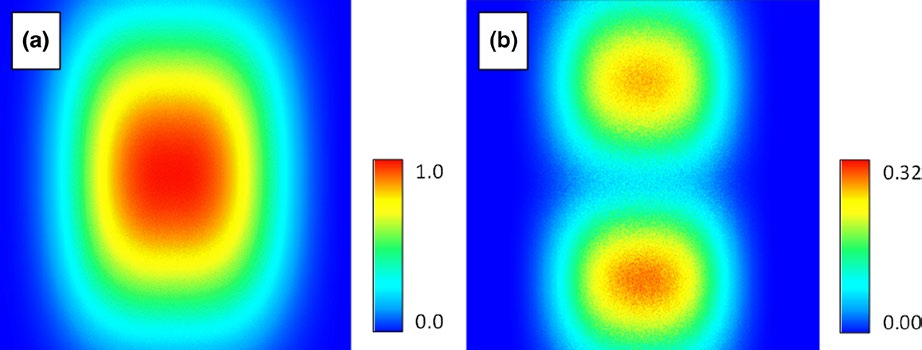
The mean predicted habitat model with normally distributed noise injected into the “BottomToTop” environmental variable (a) and the resulting uncertainty map showing the standard deviation of the habitat models generated during the run (b)

Random noise was injected into the “BottomToTop” environmental variable based on a normal distribution with a mean of 0 and a standard deviation of 10. The resulting AUC dropped to 0.76 with a standard deviation of 0.003 while the AIC rose to 26,668 with a standard deviation of 32. Noise injection resulted in the blurring of the edges of the habitat suitability map at the top and bottom of the habitat map (Figure 12). The corresponding map of standard deviation shows that uncertainty was associated with the edges of the predicted habitat suitability. The distribution of uncertainty could indicate a higher confidence in the center of the predicted habitat suitability.

**Figure 13:**
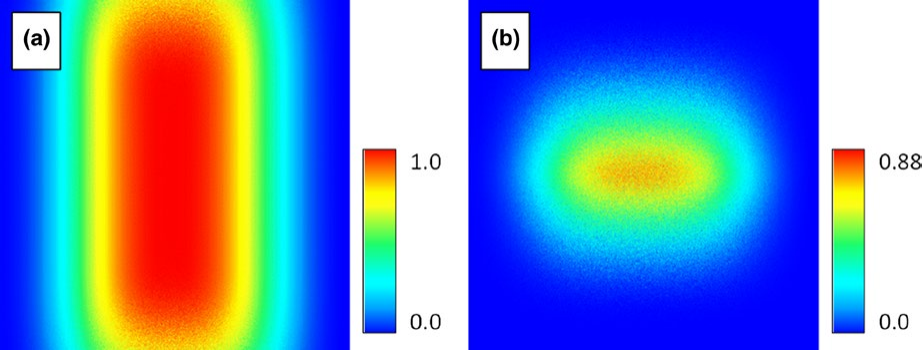
The maximum predicted habitat (a) and the minimum predicted habitat (b) when noise is injected into the BottomToTop environmental variable

The resulting maximum habitat map elongated the habitat in the direction of the BottomToTop environmental gradient. This shows the maximum potential distribution of habitat suitability among iterations for the species and could be used when managers want to maximize the conservation range for a species (Jones, 2012). An example might be when needing to survey for establishment and spread of an invasive species. The minimum habitat map shows a shrinking of the habitat in the BottomToTop direction. This map could be interpreted as having high confidence that the remaining area contains a large quantity of highly suitable habitat for the species. This might be used to set up a refuge for an endangered species (Figure 13).

**Figure 14:**
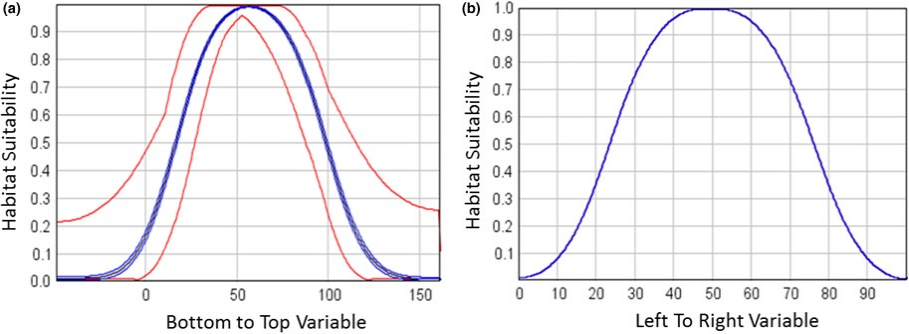
Response curves for the BottomToTop variable (a) and the LeftToRight variable (b). The resulting response curve for the unmodified LeftToRight variable is as expected while the response for the BottomToTop variable with noise injected shows a wide range of response curve values and, for the first time, we can see the 95% confidence interval around the mean response curve

The intersection of noise injected response curves extended from 10 and 90 to 0 and 100. The minimum and maximum ranges were more visible as a result (Figure 14).

## 6 MODELING SPATIALLY DEPENDENT UNCERTAINTY IN ENVIRONMENTAL VARIABLES

Available environmental variables already contain pixel values that represent a simplified mix of an area on the earth. In many cases, these homogenized landscapes are further reduced by the downsampling of environmental variables (Gottschalk et al., 2011) to reduce resolutions when modeling at large extents. Downsampling consequently amplifies the uncertainty of the pixel values. Some landscapes are, however, more variable than others such as mountainous regions (Hijmans et al., 2005). Therefore, if the pixels are very large (e.g., 1–4 km), then some individual pixels will represent a variety of habitat types (narrow canyons and plateaus) while others may be uniform (e.g., a large desert) (Hutchinson, 1991). For this reason, the spatial distribution of uncertainty from a simplified landscape is dependent on the original variability within that landscape. One solution is to obtain higher resolution data for the study area and measure the standard deviation for the pixels in the higher resolution raster that overlap with each pixel in the lower‐resolution raster. The resulting data can then be used to inject spatially dependent noise into an HSM to create maps showing areas of high certainty versus low certainty. If a higher resolution raster is not available for a specific environmental variable, another raster may be able to be used as the uncertainty. Examples might include terrain roughness for temperature variables.

To model the spatially dependent uncertainty in environmental variables, we created a synthetic habitat map with a relatively narrow area of habitat that might represent a narrow canyon. We produced 1,000 occurrence points randomly placed within the area defined as habitat. The synthetic habitat map was then used as an environmental variable with values ranging from 0 (poor habitat) to 255 (optimal habitat). Two downsampled environmental variables were created: one using nearest neighbor sampling and the other using an averaging method (Cracknell, 1998).

### 6.1 Original environmental variable

**Figure 15:**
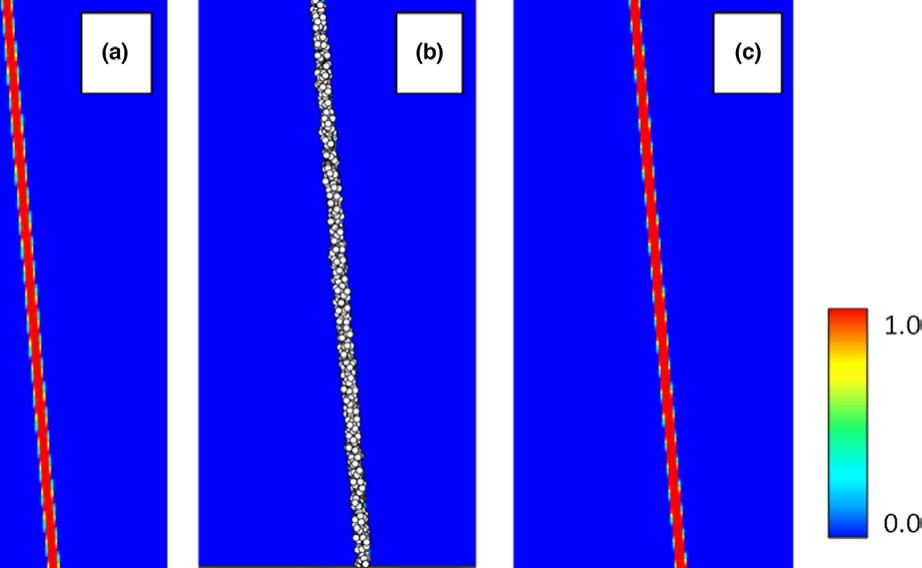
The original raster defining the available habitat for the species (a), the 1,000 occurrence points that were added based on the original habitat raster (b) and the habitat model based on the occurrences and using the original habitat as the environmental variable (c)

The model using the original environmental variable performed well with an AUC of 0.97 (AIC of 15,993) and a response curve showing the species preference for the high values (near 255) in the original habitat map (Figure 15 and X.A1).

### 6.2 Downsampled environmental variable using nearest neighbor sampling

**Figure 16:**
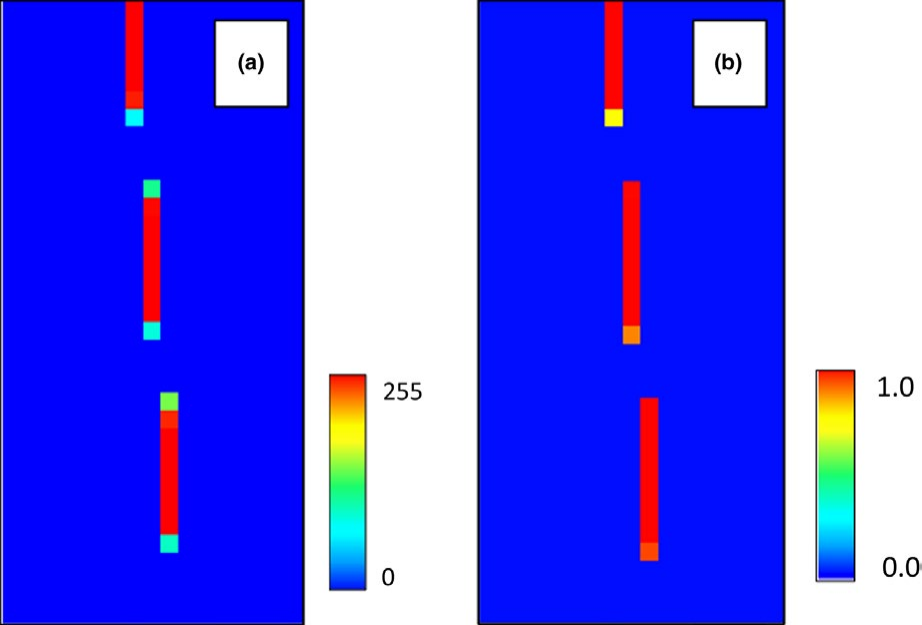
The downsampled environmental variable that now has gaps because of the nearest neighbor algorithm (a) and the resulting habitat model (b)

The next environmental variable was created by downsampling the original by a factor of 8 using nearest neighbor selection. This produced an environmental variable with breaks in the habitat. The break occurred where the nearest neighbor algorithm happened to select pixels to the left or right of the actual habitat area. This model produced an AUC of 0.78 (AIC of 10,054) and at first may appear to be an acceptable model. However, if we examine the response curve, we see that the habitat suitability has shifted to the left (Figure 19 .b1). This shows that the model includes unsuitable habitat represented by pixels that now contain occurrences because of the nearest neighbor algorithm (Figure 16).

**Figure 17:**
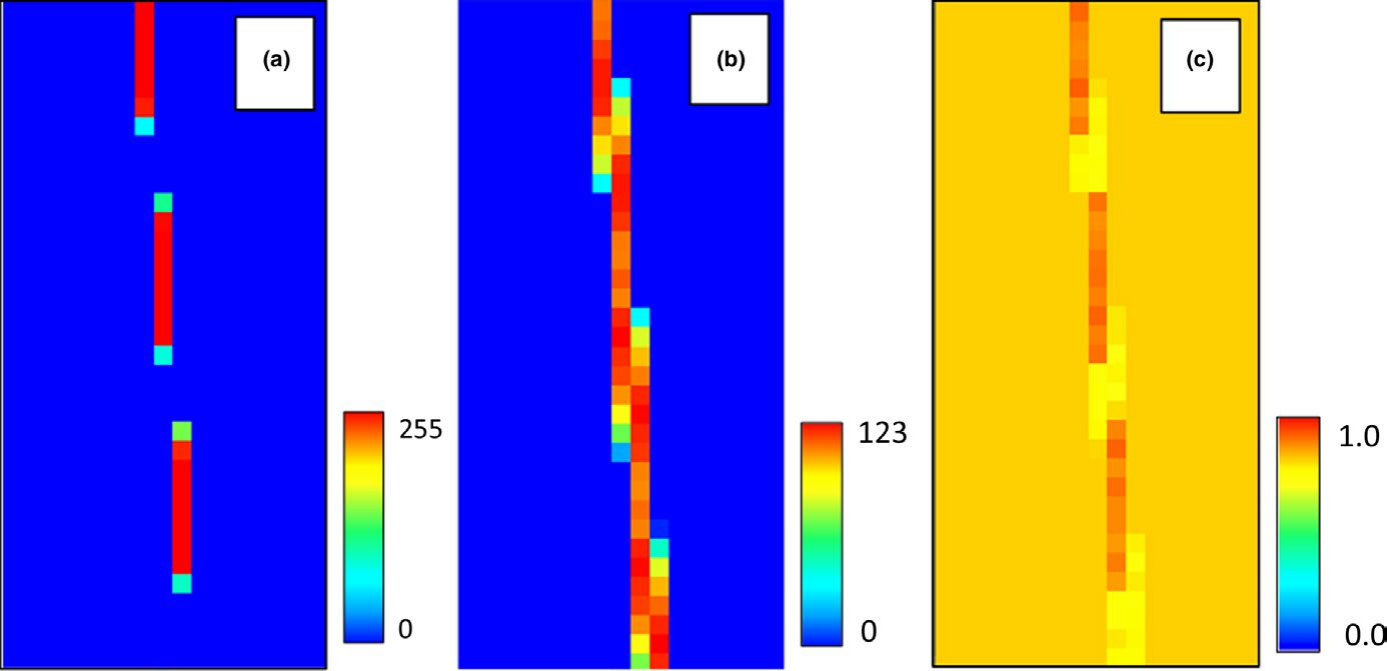
The environmental variable that was downsampled using a nearest neighbor method (a), the standard deviation of the pixels around each pixel in the downsampled environmental variable (b), and the resulting model when noise is injected into the model‐based standard deviation raster (c)

To model the uncertainty in the habitat map, we created a raster where each pixel represented the standard deviation of the neighboring pixels in an 8 × 8 grid. This raster was then downsampled by a factor of 8 to match the environmental variable. Noise was injected into the downsampled raster through the Monte Carlo feature in HEMI 2. While the inclusion of noise fully recreated the original suitable habitat, it also overpredicted the original area of the species’ habitat because of the extreme standard deviation values used (Figure 17). In a real scenario, the standard deviation values would rarely be this extreme. However, this exercise shows the value of injecting noise into models where the species habitat may be in relatively small areas. The real lesson is that the resolution of environmental variables must be high enough to maintain even small areas of habitat if the model is to be used reliably.

The resulting response curve shows a large variation in the minimum and maximum values of the response curve (Figure 19.b2).

### 6.3 Downsampled environmental variable using averaging methods

Another approach to downsampling is by averaging the pixels that overlap with the resulting pixel in the downsampled image. This method has the advantage of providing a mean of the original pixel but will also produce new values rather than using the existing values in the environmental variable raster. For this study, we created a raster that was downsampled 3 times using a bilinear averaging method (Bolstad, 2008). The resulting model had an AUC of 0.89 and a response curve that looked much like the original (Figure 19 .c1).

**Figure 18:**
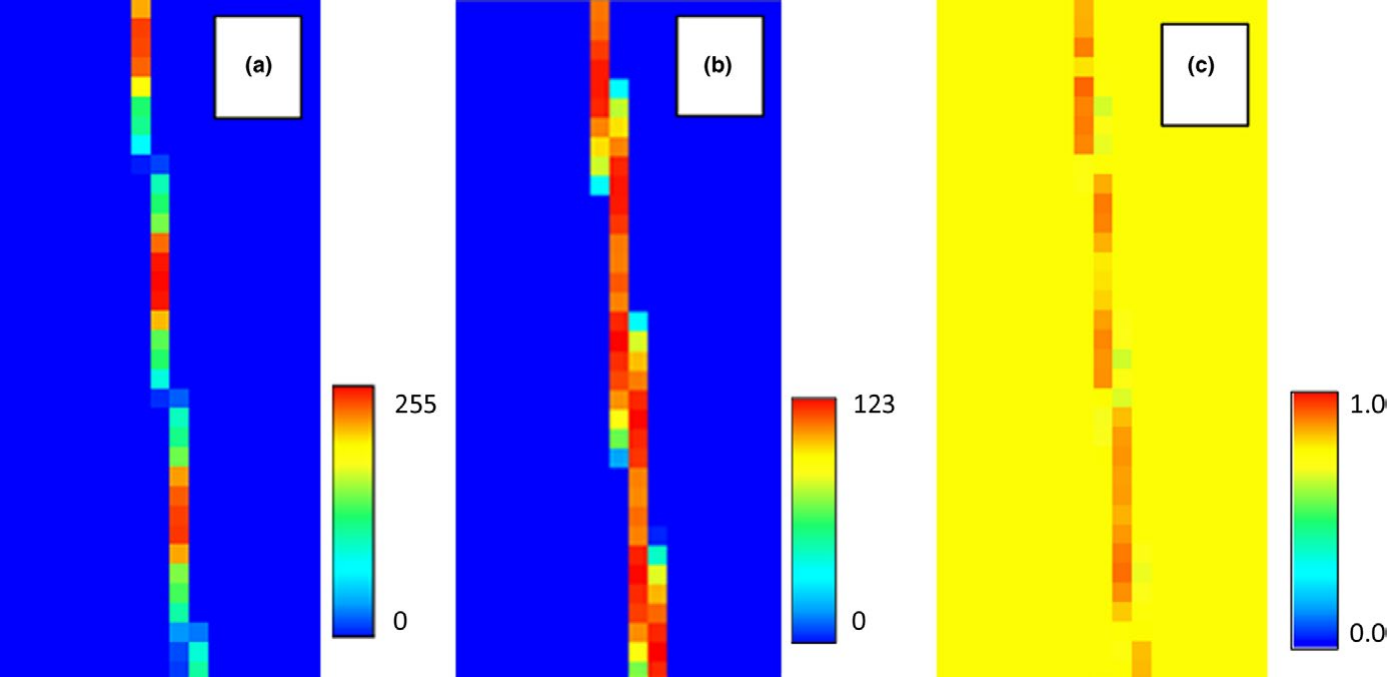
The environmental variable that was downsampled using a bilinear method applied three times (a), the standard deviation of the pixels around each pixel in the downsampled environmental variable (b), and the resulting model when noise is injected into the model‐based standard deviation raster (c)

**Figure 19:**
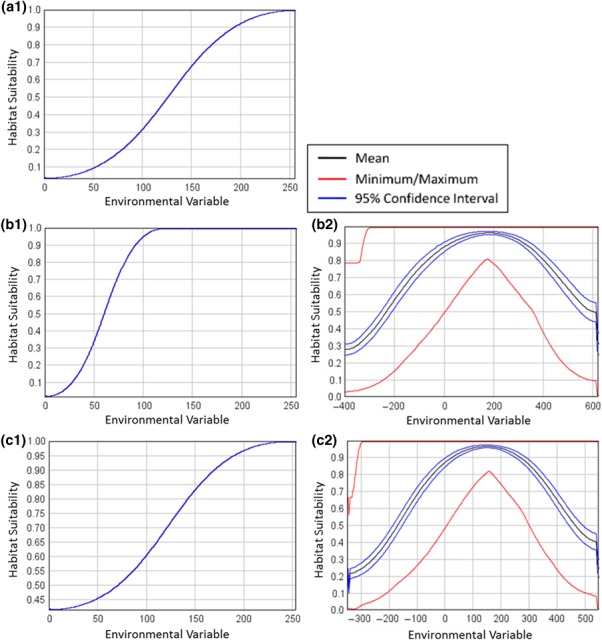
On the left are response curves based on the original environmental variable (a1), the downsampled map using nearest neighbor downsampling, (b1) and using an environmental variable that was downsampled 3 times using bilinear sampling (c1). Note that the response curve for the nearest neighbor data has shifted to the left because of the occurrence values that appear on pixels that contain values that are no longer representative of the species’ habitat. On the right are aggregate response curves of 100 models with spatially dependent noise based on the standard deviation of the neighborhood around each downsampled pixel for nearest neighbor sampling (b2) and for bilinear downsampling (c2)

The same standard deviation raster from the previous section could then be used to inject noise into the environmental variable based on the original distribution of pixels (Figure 18). This resulted in an average AUC of 0.64 and a mean response curve that was very similar to the one generated with the nearest neighbor downsampling method (Figure 19.c2).

### 6.4 Validation testing

Validation testing for the first set of synthetic data was run with 70% of the data used to train the model and the remaining 30% used for testing. 100 iterations were run and showed the model to be relatively stable, where subsets of the occurrences had a mean AIC of 8,472 and a standard deviation of 840. The mean AUC was 0.79 with a standard deviation of 0.007.

### 6.5 Sensitivity testing

Sensitivity testing of the model parameters was executed on the first set of synthetic data by injecting noise based on a mean of 0 and a standard deviation of 10 into the coefficients for the response curve for the BottomToTop environmental variable. This was accomplished by moving the control points on the response curve based on values generated from a normal distribution, which produced a mean AIC of 26,912 and a standard deviation of 852. The mean AUC was 0.79 with a standard deviation of 0.001.

## 7 CONCLUSION

With HEMI 2, we were able to create uncertainty maps of the mean, minimum, and maximum of habitat suitability by injecting noise into occurrence locations, environmental variable rasters, and model parameters. This was shown for synthetically created data including environmental variables where suitability habitat was underrepresented because of downsampling. The mean habitat suitability map can be used as an approximate measure of a species’ potential geographic range, whereas the minimum habitat suitability has the potential to provide an informed preservation guide on the most valuable habitat for a species. This may be critical when setting up a refuge for an endangered species. The maximum habitat map is useful in scenarios where we need to survey all possible areas, including small refuges, such as for an invasive species.

The noise injection features of HEMI 2 are valuable for modeling uncertainty but different features may be more valuable at different modeling extents. For small extents, injecting noise into the occurrence locations will be of value as the accuracy of data collection devices may cause occurrences to change which pixel their environmental variables are drawn from. For large extents when the pixels are large (i.e., modeling countries or the world at 1–4 km per pixel), injecting noise into the environmental variables would be of more value as the pixels represent larger areas and thus a greater variety of habitat on the ground. Typical GIS software provides the ability to find statistics for neighboring pixels when downsampling environmental variables.

Using histograms to compute model fit statistics solved the performance problems of the first version of HEMI and allowed for a larger number of environmental variables. However, the statistics varied slightly from statistics computed using the final results and the performance of the interface could still be improved. HEMI 2 was tested with over 20 environmental variables and performed well but this required the computer to have enough memory for all the environmental variables to be loaded into memory at one time. Accessing the environmental variables from files on disk would remove the memory restrictions but would also slow the performance of HEMI 2.

Injecting noise into or randomly perturbing data are recommended techniques for uncertainty testing on predictive models (Jakeman, Letcher, & Norton, 2006). The popular modeling software MaxEnt addresses uncertainty with cross‐validation, bootstrap, and jackknife testing (Elith et al., 2011). Third‐party applications for MaxEnt complement these built‐in tests with direct model comparison (ENMTools; Warren, Glor, & Turelli, 2010) and random noise injection (Gould et al., 2014), but do not provide sensitivity testing of model coefficients or a combination of all of these approaches in a single HSM. HEMI 2 is the first software package to provide a rigorous suite of uncertainty tests that include noise injection, cross‐validation, and sensitivity testing, and provides the computational strength to combine all of these in a single HSM.

The Monte Carlo methods used with HEMI 2 gave us much more confidence in our models than previous approaches. Because of this, the noise injection and validation Monte Carlo features have also been implemented for the popular SDM modeling software MaxEnt (Phillips, Dudik, & Schapire, 2004) and are available in BlueSpray.

An area of concern within HEMI 2 is that if the user provides a data set with multiple occurrences per pixel in the environmental layer, the model will shift to representing abundance and could introduce bias if the occurrence data are biased. Another concern, and one of the most controversial topics within HEMI 2, may be its ability to allow the user to move the control points of response curves. This has been shown by Kimble to be of value in some situations but should only be practiced if there is existing knowledge that specifies the species’ response to each adjusted response curve. HEMI 2, like all modeling software, is just a tool and relies on the user to make sound modeling decisions. Our goal was to provide a full suite of uncertainty tools that will increase accessibility to modelers and to provide a framework that can be later built upon. We hope that this framework and those currently available will help to provide the basis for upping the momentum of making uncertainty testing a fundamental component of habitat suitability modeling.

## 8 SOFTWARE AVAILABILITY

HEMI 2 was implemented within BlueSpray which is a geographic information system (GIS) created by SchoonerTurtles, Inc. BlueSpray is available from Humboldt State University at http://gsp.humboldt.edu/isamm/BlueSpray.html. A Quick Start tutorial is available for the modeling feature, HEMI 2, by clicking the help button (?) in the main HEMI 2 dialog. Additional tutorials and examples of HEMI 2 HSMs, including all model runs from this study and a comparison study with MaxEnt, are available at http://gsp.humboldt.edu/HEMI2.

## CONFLICT OF INTEREST

None declared.

## AUTHOR CONTRIBUTIONS

Jim Graham was the primary inventor and creator of HEMI 2 and was the primary author of the paper. Melissa Kimble contributed additions and edits throughout the paper and devised using running means to determine the number of required runs.

## DATA ACCESSIBILITY

Data presented in this paper are available at www.Pangaea.de, PDI‐17365.
